# Osseointegration Improvement of Co-Cr-Mo Alloy Produced by Additive Manufacturing

**DOI:** 10.3390/pharmaceutics13050724

**Published:** 2021-05-14

**Authors:** Amilton Iatecola, Guilherme Arthur Longhitano, Luiz Henrique Martinez Antunes, André Luiz Jardini, Emilio de Castro Miguel, Miloslav Béreš, Carlos Salles Lambert, Tiago Neves Andrade, Rogério Leone Buchaim, Daniela Vieira Buchaim, Karina Torres Pomini, Jefferson Aparecido Dias, Daniele Raineri Mesquita Serva Spressão, Marcílio Felix, Guinea Brasil Camargo Cardoso, Marcelo Rodrigues da Cunha

**Affiliations:** 1 Faculty of Medicine of Jundiaí, Jundiaí 13202-550, São Paulo, Brazil; amilton.iatecola@ceunsp.edu.br (A.I.); tiagonevesandrade@yahoo.com.br (T.N.A.); cunhamr@hotmail.com (M.R.d.C.); 2 Center for Information Technology Renato Archer (CTI), Campinas 13069-901, São Paulo, Brazil; guilonghita@gmail.com; 3 National Institute of Biofabrication (INCT-BIOFABRIS), Campinas 13083-852, São Paulo, Brazil; lhmantunes@gmail.com (L.H.M.A.); ajardini@unicamp.br (A.L.J.); beresm@metalmat.ufc.br (M.B.); 4 School of Chemical Engineering, University of Campinas (UNICAMP), Campinas 13083-852, São Paulo, Brazil; 5 School of Mechanical Engineering, University of Campinas (UNICAMP), Campinas 13083-860, São Paulo, Brazil; guinea.cardoso@ceunsp.edu.br; 6 Department of Metallurgical and Materials Engineering, Federal University of Ceará, Fortaleza 60440-554, Ceará, Brazil; emilio@metalmat.ufc.br; 7 “Gleb Wataghin” Institute of Physics, University of Campinas (UNICAMP), Campinas 13083-859, São Paulo, Brazil; lambert@ifi.unicamp.br; 8 Department of Biological Sciences, Bauru School of Dentistry (FOB/USP), University of São Paulo, Bauru 17012-901, São Paulo, Brazil; karinatorrespomini@gmail.com; 9 Postgraduate Program in Structural and Functional Interactions in Rehabilitation, University of Marilia (UNIMAR), Marília 17525-902, São Paulo, Brazil; danibuchaim@alumni.usp.br (D.V.B.); jeff.bojador@gmail.com (J.A.D.); danispressao@hotmail.com (D.R.M.S.S.); 10 University Center of Adamantina (UniFAI), Medical School, Adamantina 17800-000, São Paulo, Brazil; 11 Postgraduate Program in Law, University of Marilia (UNIMAR), Marília 17525-902, São Paulo, Brazil; 12 Veterinary Medicine School, University of Marilia (UNIMAR), Marília 17525-902, São Paulo, Brazil; felix.marcilio@hotmail.com

**Keywords:** additive manufacturing, plasma immersion ion implantation, Co-Cr-Mo alloy, osseointegration, orthopedic implants

## Abstract

Cobalt-base alloys (Co-Cr-Mo) are widely employed in dentistry and orthopedic implants due to their biocompatibility, high mechanical strength and wear resistance. The osseointegration of implants can be improved by surface modification techniques. However, complex geometries obtained by additive manufacturing (AM) limits the efficiency of mechanical-based surface modification techniques. Therefore, plasma immersion ion implantation (PIII) is the best alternative, creating nanotopography even in complex structures. In the present study, we report the osseointegration results in three conditions of the additively manufactured Co-Cr-Mo alloy: (i) as-built, (ii) after PIII, and (iii) coated with titanium (Ti) followed by PIII. The metallic samples were designed with a solid half and a porous half to observe the bone ingrowth in different surfaces. Our results revealed that all conditions presented cortical bone formation. The titanium-coated sample exhibited the best biomechanical results, which was attributed to the higher bone ingrowth percentage with almost all medullary canals filled with neoformed bone and the pores of the implant filled and surrounded by bone ingrowth. It was concluded that the metal alloys produced for AM are biocompatible and stimulate bone neoformation, especially when the Co-28Cr-6Mo alloy with a Ti-coated surface, nanostructured and anodized by PIII is used, whose technology has been shown to increase the osseointegration capacity of this implant.

## 1. Introduction

In the last decades, the rise in life expectancy, traumas, tumors, and other bone diseases increased the number of orthopedic surgeries, which demand implants with good biocompatibility, mechanical and surface properties [1]. Therefore, development of new materials and techniques is necessary to obtain more durable implants with lower rejection rates [2,3]. Thus, regenerative medicine continues to search for new scaffolds [4,5], artificial organs [6], biomaterials [7,8,9,10,11,12] and complementary therapies [13,14,15,16,17] in order to optimize tissue regeneration [18]. Among the experimental repair protocols, the use of long bones can be explained by the ease of access and surgical manipulation, as well as similarity with the clinical application in humans, regarding its remodeling, physiological repair of muscle strength and tension, which can also be analyzed in biomechanical tests [9,14,19,20,21].

Co-Cr-Mo alloys are commonly used for joint replacement biomedical implants [22,23]. However, Co-Cr-Mo alloys are hard to process by machining, which limits its production for casting techniques. In this context, additive manufacturing (AM), a layer-by-layer production technique, can be used to produce Co-Cr-Mo alloy implants [24]. Due to the nearly unlimited geometrical freedom, this technique can be employed to produce customized implants with complex geometries, considering the patient’s physical and anatomic characteristics, as well as the shape and size of the bone lesion. The AM technique allows the manufacture of a specific prototype with the same geometric characteristic of the patient’s bone lesion, thus allowing its fitting with precision and reducing the possibility of displacement and extrusion of the implant [25,26]. 

Therefore, these biomodels allow the measurement of structures, the simulation of osteotomies and resection techniques using accurate geometrical and anatomical details of a bone lesion. This leads to a reduction in the overall cost of treatment, reduced duration of surgery due to preoperative planning and, consequently, the period of anesthesia and the risks of infection. As a result, there is a significant improvement in the patient’s recovery and post-operative results [1,27]. AM techniques can be classified in 3 major categories: powder-based, extrusion-based, and vat photopolymerization. Different combinations of materials/AM techniques may be an important fact, depending on the indications for clinical applications [28]. 

Jardini et al. [1] used AM technology to design and fabricate a biomodel and customized a titanium alloy implant for a surgical reconstruction of a large cranial defect in a patient who was injured in a bike accident with a large post-trauma defect in the right-frontal bone. The large bone defect was the result of a decompressive craniotomy. The immediate outcome was successful, and the implant fitted precisely onto the large cranial defect. Therefore, the use of AM promotes reductions and cost savings for health systems and a better quality of life for a large number of patients [29,30].

In addition, the AM method allows the production of complex porous structures with controlled pore size, porosity, unit cell, and interconnectivity [31]. Co-Cr-Mo alloys present a high elastic modulus (~240 GPa) [32], which is much higher in comparison to the bone stiffness (~0.01 to 22 GPa) [31]. Therefore, the control of porosity and unit cell can be used to tailor the implant stiffness, avoiding stress shielding. Moreover, bone ingrowth and interlocking can be achieved with interconnected pores with porosities between 50% and 80% and pore sizes between 100 and 1200 µm [33,34].

Furthermore, surface modification techniques can be used to improve the implant response within the human body since the topography of the material is directly related to its ability to osteointegrate [35]. For complex geometries including porous structures, mechanical surface modifications are not suitable because of their difficulty in reaching inner parts [36]. Therefore, alternative surface modifications need to be used. Plasma immersion ion implantation (PIII) is a versatile physicochemical technique that can modify any complex surface, creating nanotopography.

Meirelles et al. [37] used PIII to modify Ti threaded implant surfaces and higher removal torques compared to turned surfaces and were measured in rabbit models. The results revealed that implant surfaces coated with Ti and modified by the PIII technique presented higher maximum loads. Sisti et al. [38] confirmed that titanium implants modified by laser irradiation can increase osseointegration during the initial phase. This information demonstrates that physicochemical and surface modifications of metal alloys are essential to accelerate the bone repair process.

Thus, it was observed that titanium, cobalt and chromium alloys are used experimentally and clinically due to their non-cytotoxicity, mechanical resistance, absence of corrosion by tissue fluid [39], and the ability to promote osseointegration [40,41]. However, these properties can be improved by the process of anodizing the surface of the material, in addition to having the possibility of being manufactured in different geometric shapes by the AM process according to the dimensions of the specific bone lesion of each patient. However, there are few studies on the use of these metallic alloys produced by AM and with ionic implantation by plasma immersion (PIII) when implanted in bone lesions. 

Based on this, the objective of the present research was to evaluate the osteoregenerative capacity of Cobalt-Chromium (Co-Cr) and Titanium (Ti) metal alloys, anodized or not, when implanted in the femur of rats.

## 2. Materials and Methods

### 2.1. Additive Manufacturing

Co-28Cr-6Mo samples were fabricated using the selective laser melting (SLM) technique. Feedstock material were commercial and included EOS Cobalt Chrome MP1 powder [EOS GmbH—Electro Optical Systems, 2011], and the EOSINT M280 AM machine from EOS GmbH. The processing parameters were the manufacturer parameters for the MP1 alloy. A nitrogen shield atmosphere was used during the production. Samples had a cylindrical geometry with 3 mm diameters and 2 mm heights. The cylinders were designed with a solid half and a porous half, with a projected 600 µm pore size and 56% porosity to allow for bone ingrowth [33,34]. 

### 2.2. Plasma Immersion Ion Implantation (PIII)

The surface of the samples was anodized using plasma immersion ion implantation. Initially, the samples were placed in a vacuum chamber that operated at 10-6 Torr. Ar gas was then purged into the chamber until 10-1 Torr was reached. Next, the samples were sputtered for 1 min by Ar plasma using a 1.5 kV tension for homogenizing and cleaning the surface. For a group of Co-28Cr-6Mo samples, a 1000 Å thick Ti layer was deposited on the surface by sputtering using pulsed tension. Finally, both non-coated and Ti-coated samples were bombarded in a 10-1 Torr Ar atmosphere using 3 kV tension. 

This final ion bombardment created a nanostructured surface because of the high-energy of the collision. Before opening the chamber, the Ar atmosphere was replaced with oxygen, generating oxygen plasma. The new atmosphere resulted in an anodized surface with a controlled oxide layer on the surface of the sample by the reaction with the oxygen ions.

### 2.3. Implant Surface Characterization

Implant surface morphology was analyzed using a field emission gun scanning electron microscope (FEG-SEM) (FEI Quanta 450 and Quanta 650), both equipped with energy-dispersive X-ray spectroscopy (EDX). Secondary electron micrographs and EDX maps were collected using a 30 µm aperture size and 20 kV accelerating voltage.

### 2.4. In Vivo Study

**Figure 1:**
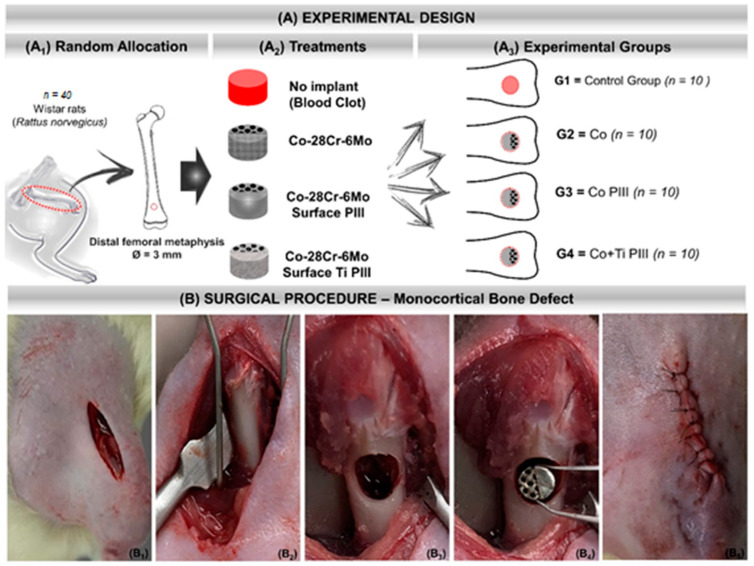
(**A**) Experimental design. (**A1**) Random Allocation—Forty adult male Wistar rats (*Rattus norvegicus*), 120 days old, weighing around 340 g. Monocortical bone defect model 3 mm in diameter in the distal metaphysis of the left femur. (**A2**) Treatments—groups according to the type of implant or not: No implant (blood clot), Co-28Cr-6Mo, Co-28Cr-6Mo with surface nanostructured by PIII and Co-28Cr-6Mo with surface coated with Ti and nanostructured by PIII. (**A3**) Experimental groups: G1 (*n* = 10)—bone defect filled with blood clot, no implant; G2 (*n* = 10)—bone defect filled with Co-28Cr-6Mo implant; G3 (*n* = 10)—bone defect filled with Co-28Cr-6Mo implant with surface nanostructured by PIII; G4 (*n* = 10)—bone defect filled with Co-28Cr-6Mo implant with surface coated with Ti and nanostructured by PIII. (**B**) Surgical procedure. (**B1**) Skin incision on the anteromedial aspect of the thigh. (**B2**) Exposure of the cortical bone of the surgical area. (**B3**) 3 mm bone defect in the distal metaphysis of the femur. (**B4**) Implantation of the metallic alloy in the surgical bed. (**B5**) Tegument suture with 5-0 nylon thread.

A total of forty male Wistar rats (*Rattus norvegicus*), 12 weeks old, male, with an average weight of 340 g, were randomly divided into four groups for in vivo experiments (Table 1). Rats were kept in cages in the bioterium of the Jundiaí Medical School, Brazil and with environmental enrichment, temperature of 22 ± 1 °C, and light/dark cycle every 12 h. They were fed with a balanced ration (Labina, Purina^TM^, São Paulo, São Paulo, Brazil) and water ad libitum. The rats were divided into four groups of 10 animals each (Figure 1A). Before the surgical procedure, the animals were individually anesthetized with a gluteal intramuscular injection of xylazine hydrochloride (Bayer^TM^ Animal Health, Porto Alegre, Rio Grande do Sul, Brazil) and ketamine hydrochloride (Ketalar, Packe-Davis^TM^, São Paulo, São Paulo, Brazil) in a 1:1 proportion and 0.1 mL/100 g of body weight. 

A sterile gauze pad soaked in a 0.9% saline solution was maintained in both animal eyes to prevent dryness of the corneas. After sterilization of the surgical field, the animals were positioned in a supine position for trichotomy of the left hind paw and local asepsis with chlorhexidine digluconate (Riohex^TM^ 2%, Rioquímica, São José do Rio Preto, São Paulo, Brazil). Tramadol (Germed^TM^, Hortolândia, São Paulo, Brazil) was applied in the gluteal intramuscularly. The surgery started with a cutaneous incision on the anteromedial aspect of the thigh, exposing the quadriceps femoris muscle. The musculature was sectioned, exposing the periosteum of the distal metaphysis of the left femur and detached from the bone to expose the cortical bone. A bone defect was drilled with a 3 mm trephine drill coupled to the pen of a mini-motor until the medullary canal was reached. 

The operated bone area was continuously irrigated with saline solution during the surgery to avoid local warming and lesions to the surrounding tissues. After creating the critical bone defect, the remaining medullary cavity was scraped and aspirated to avoid any osteogenic induction by the debris from the surgical drill (Figure 1). Table 1 summarizes the conditions and nomenclature used in the present study. The experiments were approved by the Animal Research Ethics Committee of the Jundiaí Medical School (Protocol 255/16).

The animals were euthanized six weeks post-implantation. An overdose of xylazine hydrochloride (Bayer^TM^ Animal Health, Porto Alegre, Brazil), thiopental, and ketamine hydrochloride (Packe-Davis^TM^, São Paulo, São Paulo, Brazil) in a 1:1:1 proportion of 0.2 mg/100 g of body weight was applied intraperitoneally. They were then submitted to a pneumothorax through the surgical incision of the abdominal cavity, followed by the section of the diaphragm muscle to access the thoracic cavity. After death, the left femur was removed for analysis of bone repair in the surgical area. 

From each experimental group, 5 samples were used for macroscopic, radiologic and microscopic analyses and another 5 for the biomechanical study of the operated area. For microscopic images, samples were cut and stained with Von Kossa and Stevens blue. Calcein and alizarin fluorochromatic bone stainers were used for fluorescence analysis. 

### 2.5. Macroscopic and Radiological Analyses

The removed femurs were documented with a Nikon^TM^ digital camera, model D3500 DSLR (Tokyo, Japan), assessing the presence of any abnormal condition in the surgical area. They were then radiographed with an Odel 300 mA, 100 mA focus, time of 0.06 s and 40 kV radiation and digitalized by the Agfa^TM^ system (Agfa-Gavaert Corp, Mortsel, Belgium) for a radiographic analysis of the surgical area.

### 2.6. Fluorescent Labeling

The Alizarin red and Calcein dyes were prepared with disodium phosphate and saline solution. For each experimental group, five animals received these fluorochromes subcutaneously on the back during the postoperative period. Alizarin Red S 30 mg/kg injections (Sigma-Aldrich^TM^, Merck KGaA, Darmstadt, Germany) were performed in the immediate postoperative period and 7 days after the surgical procedure. Calcein 10 mg/kg (Sigma-Aldrich^TM^, Merck KGaA, Darmstadt, Germany) injections were performed at 14 and 21 days. The samples were fixed in 10% buffered formaldehyde, dehydrated in increasing concentrations of alcohols, embedded in glycol methacrylate resin and polymerized.

The coronal and semi-serial histological sections of 200 μm thicknesses were made and reduced with the aid of an automatic sander (2300 rpm speed) to 30–50 μm by the Exakt CuttingGrinding System (Exakt^TM^ Apparatus GmbH, Norderstedt, Germany). The sections were analyzed using a TCS SP5AOBS laser scanning confocal microscope (LeicaTM, Wetzlar, Germany), coupled with a DFC 310 FX camera (Leica^TM^, Wetzlar, Germany), and QWin 3.1 (Leica^TM^, Wetzlar, Germany). The excitation lasers were 488 nm (calcein) and 543 nm (alizarin red).

### 2.7. Von Kossa Staining 

Von Kossa staining was applied to verify extracellular matrix mineralization in newly formed bone tissue, such as calcium and potassium ions. The preparation of slides followed the same protocol as the anterior confocal analysis. The slides were washed three times in distilled water, immersed in 1% silver nitrate solution under ultraviolet radiation for 1 h and after were immersed in 5% sodium thiosulfate solution and immediately washed in distilled water. 

This process gives the pink and black colorations, referring to the cytoplasm and calcium ions, respectively. Cell nuclei were counterstained by immersion for 5 min in safranin solution and glacial acetic acid. The slides were observed using a Motic BA310E series optical microscope (Motic^TM^, Kowloon, Hong Kong).

### 2.8. Stevens Blue Staining 

Stevens blue staining was applied to verify the morphology and topography of newly formed bone tissue in the surgical area. The preparation of slides for this method followed the same protocol as the confocal laser analysis, and the sections were stained with Stevens blue and assessed using a light Motic BA310E series optical microscope (Motic^TM^, Kowloon, Hong Kong).

### 2.9. Biomechanical Tests

**Figure 2:**
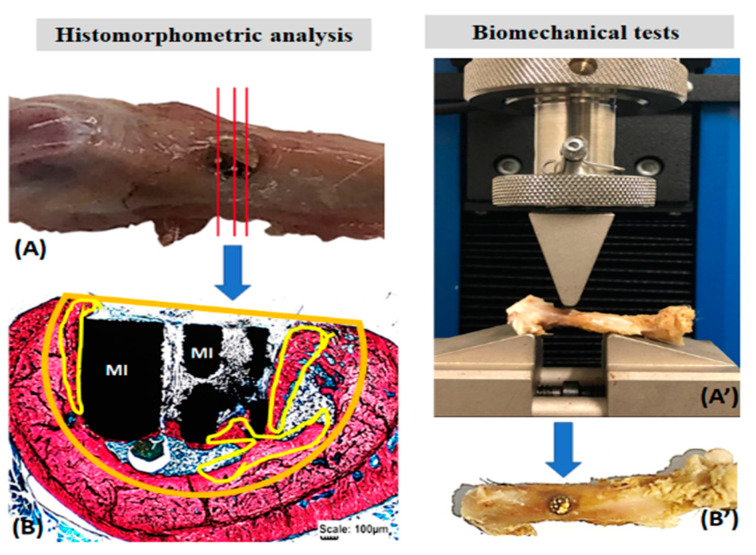
Histomorphometric analysis of the formed bone volume (**A**,**B**) and the biomechanical resistance of the surgical area in the femur of rats (**A’**,**B’**). Histological sections of the total surgical area (**A**) stained with Stevens blue to delimit the volume of the bone defect (orange) and the formed bone (yellow) (**B**). In biomechanics (**A’**), the surgical area (arrows, (**B’**)) was subjected to a load by the rod of the EMIC 23-2S equipment (**A’**). Metallic implant (MI).

The removed femurs were thawed at room temperature for the biomechanical tests and kept in saline solution until testing. An Instron EMIC 23-2S universal testing machine fitted with a 1 kN load cell was used. A pre-load of 5 N was used. The load was applied by a 3 mm cylindrical rod at 2 mm/min speed, perpendicular to the longitudinal axis of the surgical area in the anteroposterior direction until fracture (Figure 2). 

### 2.10. Histomorphometric and Statistical Analysis

Stevens blue images were used to quantify the newly formed bone volume using the Motic Images Plus 2.0 software (Motic Digital Microscopy^TM^, Kowloon, Hong Kong). In the histological sections, the total area of the bone defect was delimited, as well as the newly formed bone. Thus, the proportion in percentage of bone volume formed in the surgical area was obtained (Figure 2). Data were transcribed to the BioEstat 5.3^TM^ software, applying the ANOVA tests followed by the Tukey’s test for statistical evaluation, obtaining means and standard deviations between the study groups with a significance level set at *p* < 0.05.

## 3. Results

### 3.1. Surface Characterization

**Figure 3:**
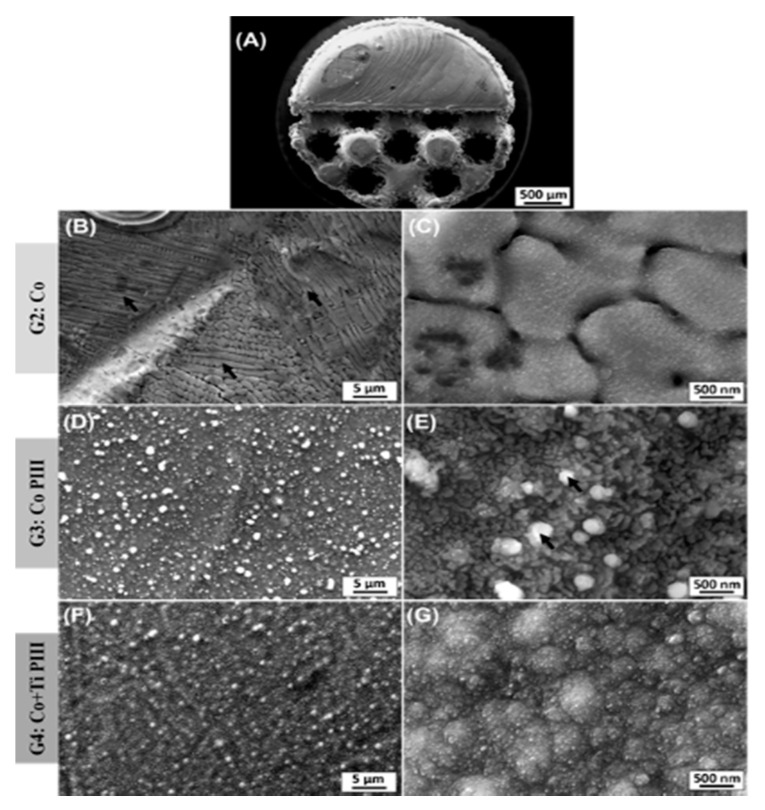
Scanning electron microscope (SEM) images of various surface finishes. Overall view from the as-built AM implant (**A**). As-built surface (**B**,**C**). Nanostructured and anodized by the PIII surface (**D**,**E**). Coated with Ti and nanostructured and anodized by the PIII surface (**F**,**G**). Black arrows in (**B**) indicate dendritic arms and in (**E**) spherical oxides.

The description of the results of the surface characterization was based on scanning electron microscope (SEM) images and energy-dispersive X-ray spectroscopy (EDX) results for the as-built surfaces and after PIII (Figure 3 and Table 2). An overview of Co-28Cr-6Mo implants made by AM with solid and porous halves was obtained (Figure 3A). The as-built material composition (Table 2) is in accordance with the manufacturer’s material datasheet (EOS GmbH—Electro Optical Systems) and with the specifications to be used as a surgical implant (ASTM F75-12). Microstructures composed of cellular dendrites are formed during the SLM building process (Figure 3B,C), where the molten material is submitted to high cooling rates and non-equilibrium structures are obtained [24,42]. It is possible to verify the nanostructures anodized by the PIII surfaces (Figure 3D,E). The high oxygen concentration from the EDX analysis reveals that the surface is covered by oxides formed by the reaction of oxygen plasma with the matrix elements during the PIII process. For the Co PIII condition, the oxides show a homogeneous morphology with some spherical oxides (Figure 3E). EDX elements concentration maps revealed that no preferential composition for the spherical oxides was found. Finally, the surface of the Co + Ti PIII condition is also covered by oxides, and the EDX analysis shows the presence of oxygen and titanium (32.98 ± 0.12 wt %), in its composition (Figure 3F,G). In this case, the presence of titanium is responsible for an oxide layer with a different morphology.

### 3.2. Macroscopic and Radiological Analyses of the Surgical Area in the Femur

**Figure 4:**
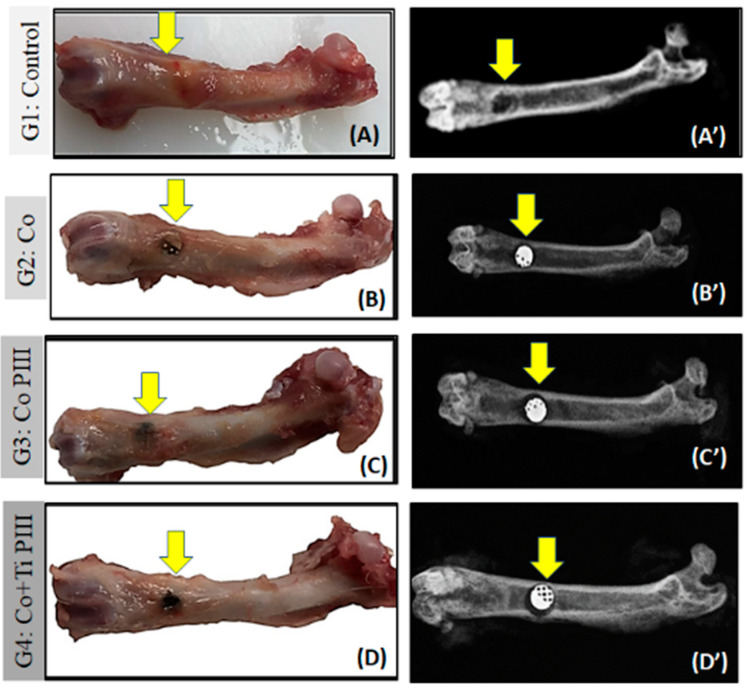
Evaluation of macroscopic features (**A**–**D**) and X-ray (**A’**–**D’**) of defects in the femur bone rats (arrows). Groups: G1: Control—no implant; G2: Co—Co-28Cr-6Mo alloy; G3: Co PIII- Co-28Cr-6Mo with surface nanostructured by PIII; G4: Co+Ti PIII—Co-28Cr-6Mo alloy surface coated with Ti and nanostructured by PIII. Macroscopic images do not show any focus of infection or inflammatory process. The radiographic images show correct positioning of the metallic implants (MI) in the G2, G3, G4 groups as well as their radiodensities.

In the surgical area of all animals, there were no pathological changes such as exacerbated inflammation and no corrosion or displacement of metallic implants (MI). All femurs presented a good bone integrity, conformity and tissue repair without infection. The radiographic images show a well-delimited bone defect and the correct positioning of the metallic implants. Furthermore, no signs of osteonecrosis, bone rarefaction or periosteal reactions were found (Figure 4). 

### 3.3. Histological Analysis of the Surgical Area in the Femur

**Figure 5:**
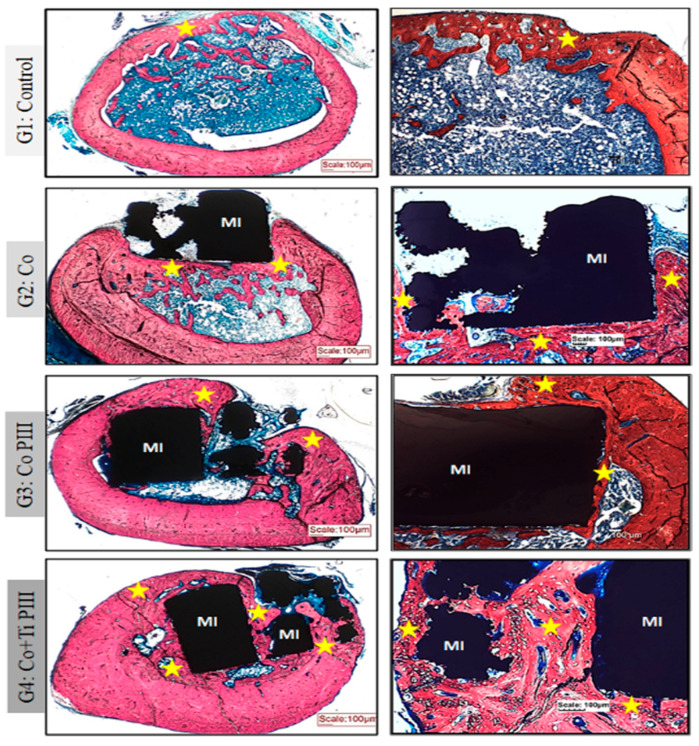
Evaluation of histologic features of defects in the femur bone of rats, Stevens blue stain. Groups: G1: Control—no implant; G2: Co—Co-28Cr-6Mo alloy; G3: Co PIII—Co-28Cr-6Mo alloy with the surface nanostructured by PIII; G4: Co+Ti PIII—Co-28Cr-6Mo alloy with the surface coated with Ti and nanostructured by PIII. All groups show bone neoformation (yellow star) from the defect margins being more intense in G4 since it involves most of the metallic implant and proportionally with a lower volume of connective tissue, essential for osseointegration. Metallic implant (MI). Bar: 100 µm.

**Figure 6:**
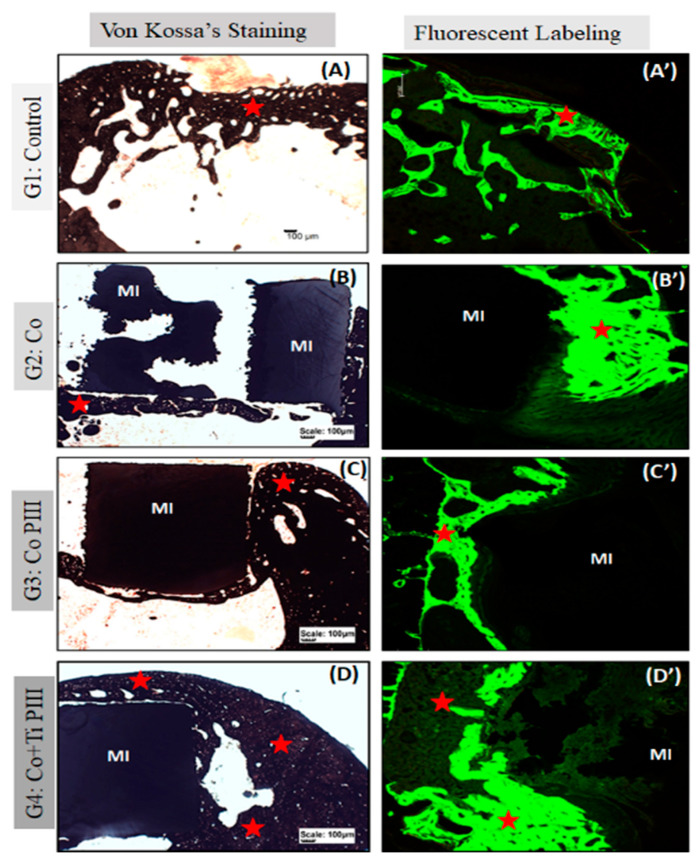
Evaluation of histologic features of defects in the rat femur bone, Von Kossa’s Staining under conventional light microscopy (**A**–**D**) and Fluorescent Labeling under Confocal Laser Scanning Microscopy (**A’**–**D’**). Groups: G1: Control—no implant; G2: Co—Co-28Cr-6Mo alloy; G3: Co PIII—Co-28Cr-6Mo alloy with surface nanostructured by PIII; G4: Co+Ti PIII—Co-28Cr-6Mo alloy with surface coated with Ti and nanostructured by PIII. All groups show bone neoformation (red star) from the defect margins being more intense in G4 since it involves most of the metallic implant and proportionally with a lower volume of connective tissue, essential for osseointegration. The Von Kossa technique demonstrated the mineralization of newly formed bone. Fluorescence was similar for all groups with new bone formation from the margins of the lesion, and there is a predominance of green over red. Metallic implant (MI). Bar: 100 µm.

Stevens blue (Figure 5) stain showed no fibrous tissue or granuloma for all groups. Furthermore, all groups show bone neoformation from the defect margins, which is mineralized as shown by the Von Koss stain and permeated by fluorophore stain (Figure 6).

For G1, subperiosteal bone formation with a trabecular aspect united the margins of the bone defect. The subperiosteal bone thickness was smaller than the original cortical bone and the trabeculae cavities were filled with hematopoietic tissue from the medullary canal. For G2, a trabecular neoformed bone was formed but limited to the lateral and inferior margins, and the osseointegration process was initiated. The bone defect was only partially closed. For G3, the implant was housed in the medullary canal and trabecular neoformed bone occurred in the lateral and upper part of the implant. The osseointegration process was initiated; however, connective tissue was formed in the lower part of the implant, and the bone defect was not closed. Finally, for G4, the trabecular neoformed bone showed some regions with lamellar bone tissue, indicating cortical bone formation, and no connecting tissue was formed between the bone and the implant surface, indicating a good osseointegration. In addition, almost all medullary canals were filled with neoformed bone, and the pores of the implant were filled and surrounded by bone ingrowth, thus demonstrating the best bone regeneration results for G4.

### 3.4. Histomorphometric Evaluation of Formed Bone Volume and Biomechanics of the Surgical Area in the Femur

**Figure 7:**
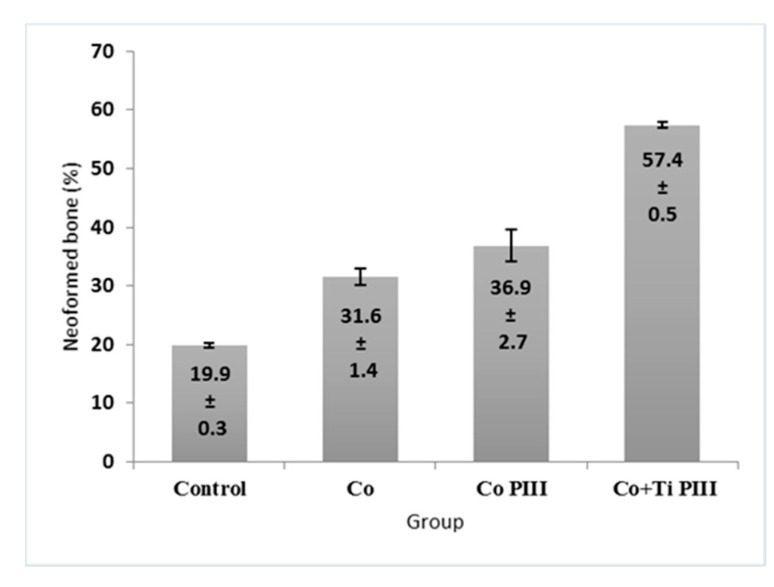
Percentage (%) of newly formed bone in the studied groups. G1 (Control), G2 (Co), G3 (Co PIII), G4 (Co+Ti PIII).

**Figure 8:**
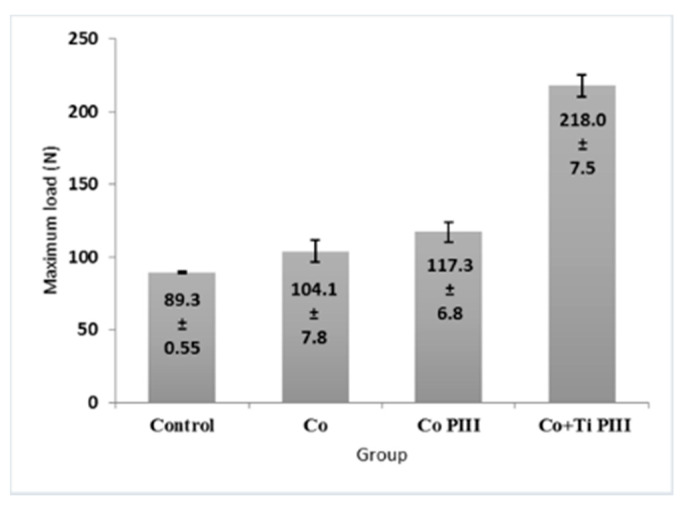
Maximum load of mechanical resistance of the surgical area in the studied groups. G1 (Control), G2 (Co), G3 (Co PIII), G4 (Co+Ti PIII).

The morphometric measurements of neoformed bone and maximum load obtained in the biomechanical tests were evaluated (Figure 7 and Figure 8, respectively). For the morphometric measurements, G1 presented the lowest neoformed bone percentage, while G2 and G3 presented medium values without statistical differences, and G4 presented the highest percentage of new bone. For the biomechanical tests, G4 presented the highest maximum load, promoted by a robust bone interlocking as shown by the higher percentage of neoformed bone and bone ingrowth in the implant pores. For the other groups, G2 and G3 again presented no statistical differences between their values; however, G3 presented a statistical difference from G1 (*p* < 0.05).

## 4. Discussion

Metallic biomaterials are often used as a substitute for injured bone and joint parts in various situations such as trauma, tumor resections, osteotomies, congenital deformities and peripheral degenerative osteoarticular diseases [1,30,43,44]. The surgical use of this type of metallic material will depend on its cost and ease of manufacture to meet the high demand for surgery, as well as on its structural characteristics such as toughness, elasticity, resistance to mechanical loads, being anticorrosive and with a controlled surface to avoid release of its components and ions into the receptor tissue [1,30]. According to Santiago et al. [45], the surface properties of an implant are fundamental for its clinical application since bone cells can recognize and respond differently depending on the structure of the implants.

The surface of an implant has a direct influence on its anchoring to the bone [46,47]. It is responsible for direct contact with the patient’s tissues, being the primary factor for osseointegration, protein adsorption, interaction with cells, the creation of the interface between the bone and implant, as well as for the development of regenerated tissues. Topography, chemical characteristics, load and wettability are some of the properties that relate the implant surface to its osseointegration capacity [46,48]. Other characteristics are also fundamental in metal alloys, such as roughness and the ability to be manufactured in different shapes so that the geometry and extent of the patient’s bone injury can be adapted [1,30].

In the present research, metallic alloys with rough surfaces, nanostructured and anodized by PIII were used, as in the cases of the G3 and G4 experimental groups, which showed better results in the process of repairing bone damage caused in animals. According to Kulkarni et al. [49], the behavior of bone cells is much more influenced by nanostructures (1 to 100 µm) than by microstructures (1 to 100 µm).

Porous coatings, layers and rough surfaces with osteoconductivity and osteoinductivity are essential qualities of implants during the osseointegration process [47]. Thus, it is necessary to use specific techniques for manufacturing customized metal alloys so that you can control the topography of the material according to the patient’s needs. This includes the size, shape and mechanical properties of the bone lesion area so that the biomodel is made with the same and precise dimensions. For this, there is the technology of additive manufacturing (AM) that is capable of producing biomodels customized according to the needs of patients and that quickly promotes their osseointegration during the post-surgical period [1].

This technology can be used in several medical fields, including neurosurgery, maxillofacial surgery, plastic and craniofacial surgery, orthopedics and dentistry, in dermatology, scaffolding and in many other areas, including even veterinary sciences [6,44,50,51,52]. Furthermore, it allows the production of three-dimensional biomedical prototypes that accurately represent the anatomy of the patient’s bone lesion. Thus, prior surgical planning can be done to facilitate procedures and avoid complications and risks during and after surgery to implant the metal alloy in the patient’s bone defect [53,54].

The main advantages of AM include the ability to produce complex geometries and minimize operator intervention, having only one step and equipment responsible for the production of the part and the time and cost of relatively small manufacturing [55]. In addition, it allows the production of porous structures with control of the geometry and size of the pores, an important fact for use in orthopedics, as they can facilitate the control of stiffness, avoiding the phenomenon of bone resorption. As a result, it provides the optimization of the implant fixation in the bone, reducing the implant adaptation time in the tissue and increasing its durability [35,56]. Disadvantages are the fact that the mechanical properties are not the same as the materials manufactured by conventional methods (because the material is made in layers, the material has anisotropy), the precision and surface finish of the parts are lower than the parts produced by machining, and the prices of the machines are high [52,57].

In the present research, metallic alloys produced by AM were used so that they could present a structure suitable for the process of osseointegration and biocompatibility when implanted in defects experimentally caused in the femur of rats. The alloys chosen were cobalt-chromium and titanium, because they meet the mechanical and chemical requirements of the ASTM F 136 standard for surgical applications, as well as stainless steel alloys [43,47,58]. According to Sisti et al. [38], titanium has excellent biocompatibility and resistance to corrosion.

Biocompatibility is one of the main aspects to be considered in the process of using implants and is related to the ability of a material to trigger an adequate response from the host during a specific application [59]. According to Billström et al. [60], any type of biomaterial as bone support must not only maintain, induce and restore biological functions, but also have the right characteristics with regard to non-immunogenicity. Shors [61] added that the successful implantation of materials is related to the viability of the receiving site in allowing bone growth and implant stability, without the presence of macro movements that can interfere with the process of bone consolidation and implant fixation.

The degradation of metallic implants in the human body can compromise the integrity of the material and its biocompatibility by causing inflammation or allergic reactions [62,63], and the derived debris can enter the bloodstream. Thus, metallic biomaterials must be resistant to corrosion and wear and tear caused by fatigue. Meeting these requirements, titanium is suitable because it has a high strength/weight ratio, non-magnetic properties and high resistance to corrosion due to the formation of a compact protective film on the titanium oxide (TiO_2_) metallic surface [1,30].

The biocompatibility characteristics described above were observed in the results of the present research using Cobalt, Chromium and Titanium alloys, implanted in rat femoral defects since the macroscopic and radiological data did not show any type of degradation of the metal alloys and also did not present infectious pathological alterations that could suggest incompatibility between the bone and metal alloys. Another result that supports this biocompatibility is the fact that there was no displacement or extrusion of the metal alloys from the implanted site. In the histological findings, no chronic inflammatory exudates were observed that could suggest the presence of infectious reactions resulting from an immunological rejection of the metal alloys implanted in the groups of animals that received these materials, in this case, G2 to G4. In addition, no signs of corrosion of the metal implants were observed since the release of ions could result in several biological reactions that could compromise the entire process of accepting the material by the receiving tissue as well as its interaction with the bone [64,65]. 

A study by Depprich et al. [66] evaluated the corrosion resistance of some metal alloys, concluding that the elements of the composition of the alloys such as iron, copper and silver decrease their corrosion resistance and are also more prone to oral pH variation. Noble metals, such as cobalt and molybdenum, increase corrosion resistance. Among the tested alloys, titanium and nickel-chromium were the most resistant to corrosion. The corrosion resistance of titanium is very high due to the fact that its surface is covered by some atomic layers of titanium oxide, which in turn is stable and, even if removed, it forms again thus preventing corrosion, which guarantees implant stability [67].

Ganbold et al. [68] evaluated the viability of cell culture in 3D-printed Co-Cr alloys and manufactured using the selective laser fusion method (SLM) and had good results from these alloys comparable to those of the conventional casting method. This proves that not only the ionic constitution, but also the manufacturing method of the metal alloys, are directly related to the interaction of the implant with the recipient tissue. Other studies support this information because they find an absence of immunological reactions and, in the case of titanium, it is due to the dense and well-adhered oxide layer that is formed in contact with different environments, such as air or water [29,35,69,70,71,72]. Other researchers claim that the stable and protective oxide layer helps to connect the extracellular matrix to the implant surface [73,74].

In some cases, in addition to the biocompatibility properties, another factor of interest is the ability to osseointegrate, which is the anchoring of an implant to live bone, obtained through the contact between them, where the migration of bone cells to the implant surface occurs, such anchorage being stable and lasting, allowing the transmission of efforts to neighboring tissues [46].

The surface of the material is essential for osseointegration [24,44,47,75]. Within this concept, the production of metal alloys with micro-roughness on the surface (1 to 10 µm) can be done by sandblasting, acid attack, plasma spray and other technical modification surfaces [76,77,78,79], as well as by AM, which in turn allows design control, customized porosity and roughness [80,81]. Research has shown that adhesion, growth and osteoblastic cell differentiation are favorable to the integration of the implant with bone tissue and are related to surface energy and roughness [40,76]. Other research has also highlighted the influence of the porosity of metal alloys on bone growth by facilitating the movement of body fluids, as well as cell and vascular proliferation through pores [82,83,84,85], thus allowing stable implant fixation through osseointegration [86,87].

In the present research, metal alloys manufactured by AM (G2, G3, G4) and with projected pores of 600 µm and 56% porosity were used because they are suitable for allowing bone growth [33,34]. These alloys were bombarded with ions in an air atmosphere at 10^−1^ Torr and with a voltage of 3 kV, creating a nanostructured surface because of the high energy of the collision. In addition, the air atmosphere was replaced by oxygen, resulting in an oxide layer controlled on the sample surface by the reaction with the oxygen ions, thus preventing the migration of the ions to the recipient bone tissue. These structural characteristics of metal alloys, promoted by the manufacturing technique of AM and immersion in plasma in the present research, contributed to bone formation in all cases where the alloys were implanted in the femoral defect caused in rats, however, with different volume and mechanical resistance between the groups, as demonstrated in the histomorphometric and biomechanical analyses of the present research.

In the metal alloys used in the present study, there was a proliferation of new bone formation on the surface of the materials, especially in the G4 group (Co-Cr-Ti), where there was a greater bone volume in contact with the recipient bone, without interposition of connective tissue, featuring osseointegration right at the beginning of bone repair, as demonstrated by the fluorescent marking technique under confocal laser scanning microscopy. Furthermore, the G4 showed greater biomechanical resistance in the implanted area compared to the other experimental groups. Thus, the titanium used in G4 can be considered, in addition to being biocompatible, a determining factor in the bone regeneration process with regard to the volume and mechanical strength of the bone formed. Titanium is a light non-ferrous, non-magnetic metal and is known for high mechanical strength values associated with low density [1,47]. Among titanium alloys, the most used in manufacturing of surgical implants is the Ti6AI4V. The structure of this alloy is a type ϸ where the stabilizing elements are aluminum and vanadium [88].

In the G2 and G3 groups, in which the anodized Cobalt-Chromium and Cobalt-Chromium alloys were used, respectively, bone neoformation also occurred, but in a smaller amount in relation to G4. In G2, connective tissue was observed at the bone implant interface, unlike G3 in which there was direct contact of the bone formed with the alloy. This indicates that the anodizing process of Cobalt-Chromium (G3) alloys may have influenced better osseointegration when compared with G2, where the same alloy was used, but not anodized. However, all the alloys used had satisfactory results in stimulating bone formation when considering that the bone volume formed in the groups with metallic implants (G2, G3, G4) was higher than that found in the G1 control group. Thus, although the Co-Cr-Ti alloy has shown better results for bone repair, it must be considered that all metal alloys used in the present research, and which were manufactured by additive manufacturing, may receive attention in new studies of bioengineering applied in regeneration experimental bone [24,42].

Additional data from future research on these alloys produced by SLM through AM may reinforce the effectiveness of these biomodels in medical and dental applications during complex bone reconstruction surgeries where there is a need for planning and surgical precision in addition to the use of customized prototypes according to the anatomical geometry of the bone lesion and the patient’s physical characteristics.

As a final consideration, the objectives, the methodology used and the results obtained in this experimental protocol make it possible to be included in the scope of the special issue of the scientific journal Pharmaceutics “Emerging Strategies to Improve the Design and Manufacturing of Biocompatible Therapeutic Materials”.

## 5. Conclusions

It was concluded that the metallic alloys of Co-28Cr-6 produced by AM are biocompatible and stimulate bone neoformation in critical defects experimentally caused in the femur of rats. However, the best results in bone quantity and biomechanical quality were of the Co-28Cr-6Mo alloy with a Ti coated surface, nanostructured and anodized by PIII, whose technology has been shown to increase the osseointegration capacity of this alloy during the initial phase of the bone repair process. Therefore, this metallic alloy (Co+Ti PIII) can be a good alternative in the use for the clinical treatment of patients with compromised bone conditions.

## Figures and Tables

**Table 1:** Summary of conditions and nomenclature adopted for each group of implants.

Group	Condition
G1: Control	Control group—no implant
G2: Co	Co-28Cr-6Mo made by AM (as-built)
G3: Co PIII	Co-28Cr-6Mo made by AM with surface nanostructured and anodized by PIII
G4: Co+Ti PIII	Co-28Cr-6Mo made by AM with surface coated with Ti, nanostructured and anodized by PIII

Co: cobalt; PIII: plasma immersion ion implantation; Ti: titanium.

**Table 2:** Surface chemical composition obtained from the energy-dispersive X-ray spectroscopy (EDX) analysis.

Group	Elements (Weight Percent—wt %)						
	Co	Cr	Mo	Si	Mn	O	Ti
G2: Co	63.04 ± 0.10	28.66 ± 0.07	6.55 ± 0.09	1.03 ± 0.03	0.73 ± 0.04	-	-
G3: Co PIII	53.14 ± 0.13	25.9 ± 0.09	5.07 ± 0.10	0.56 ± 0.02	0.62 ± 0.04	14.71 ± 0.15	-
G4: Co+Ti PIII	29.47 ± 0.12	13.72 ± 0.08	2.53 ± 0.08	0.3 ± 0.02	0.43 ± 0.05	20.58 ± 0.2	32.98 ± 0.12

Co: cobalt; PIII: plasma immersion ion implantation; Ti: titanium; Cr: chrome; Mo: molybdenum; Si: silicon; Mn: manganese; O: oxygen; Ti: titanium.

## Data Availability

The data presented in the present study are available on request from the corresponding author. The data are not publicly available because they are part of a doctoral thesis not yet deposited in a public repository.
